# Supplementary figures and images for: JAK/STAT mediated insulin resistance in muscles is essential for effective immune response
Source: Cell Commun Signal. 2024 Apr 2;22:203. doi: 10.1186/s12964-024-01575-0 (PMC10986132; doi:10.1186/s12964-024-01575-0)

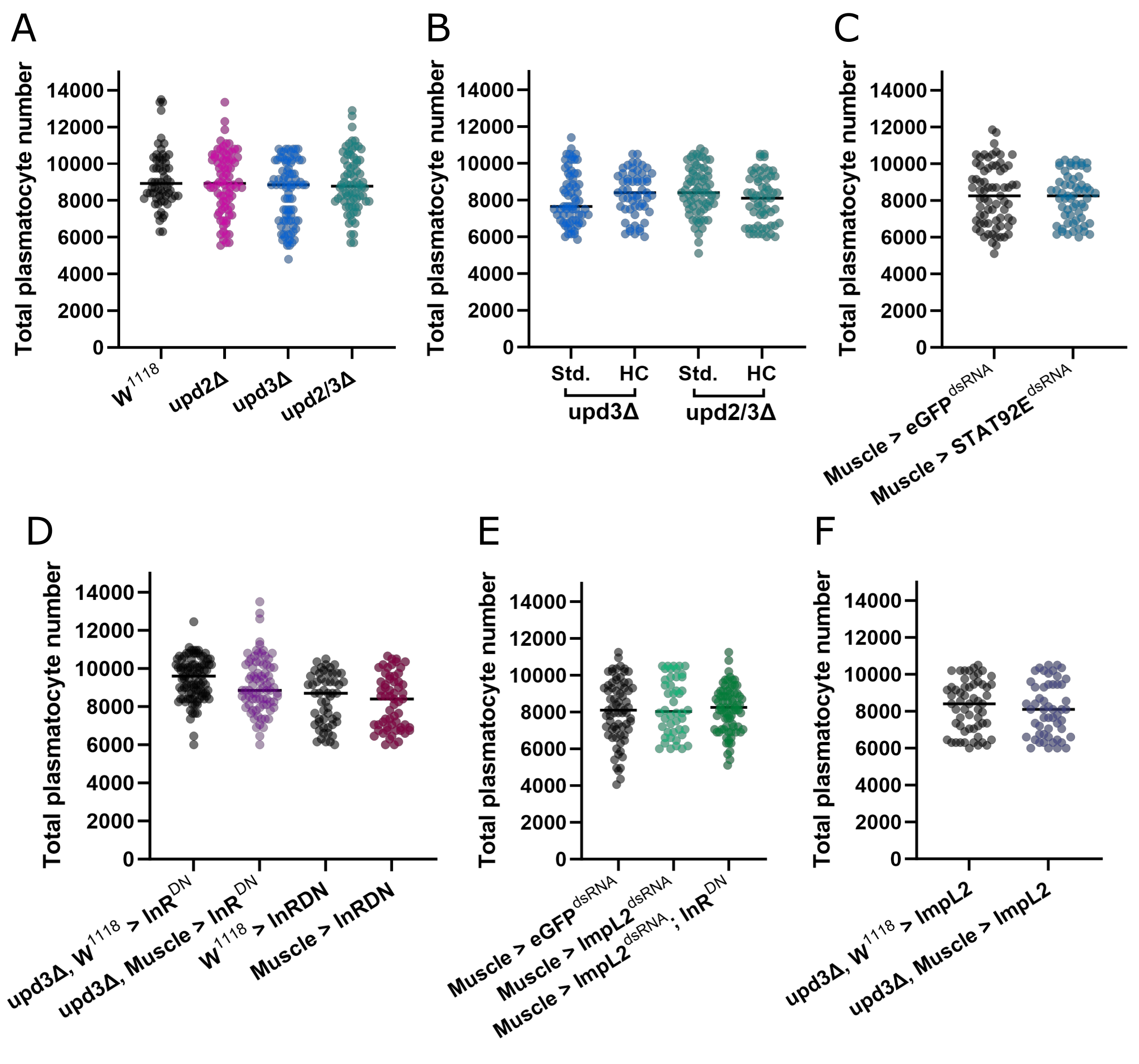

Supplement: Supplementary file 1 — Supplementary Material 1: Supplementary Fig. 1 Plasmatocyte number is not effected by knockout or knockdown of Upd/JAK/STAT signaling. Plasmatocyte count at 22 hpi for the following genotypes A: w1118 (control), upd2Δ, upd3Δ and upd2/3Δ (N = 3, n = 62–85). B: upd3Δ and upd2/3Δ on standard and high carbohydrate diet (N = 3, n = 56–67). C: Muscle > eGFPdsRNA and Muscle > STAT92EdsRNA (N = 3, n = 108–120). D: Upd3Δ, W1118 > InRDN, Upd3Δ, Muscle > InRDN, W1118 > InRDN and Muscle > InRDN (N = 3, n = 76–79). E: Muscle > eGFPdsRNA, Muscle > ImpL2dsRNA and Muscle > ImpL2dsRNA InRDN (N = 3, n = 48–71). F: Upd3Δ, W1118 > ImpL2 and Upd3Δ, Muscle > ImpL2 (N = 3, n = 53–58). Each dot represents number of lamellocytes from an individual larva. There is no significant difference in the number of plasmatocytes. N represents individual experiments, n represents biological replicates. [file 12964_2024_1575_MOESM1_ESM.tif]

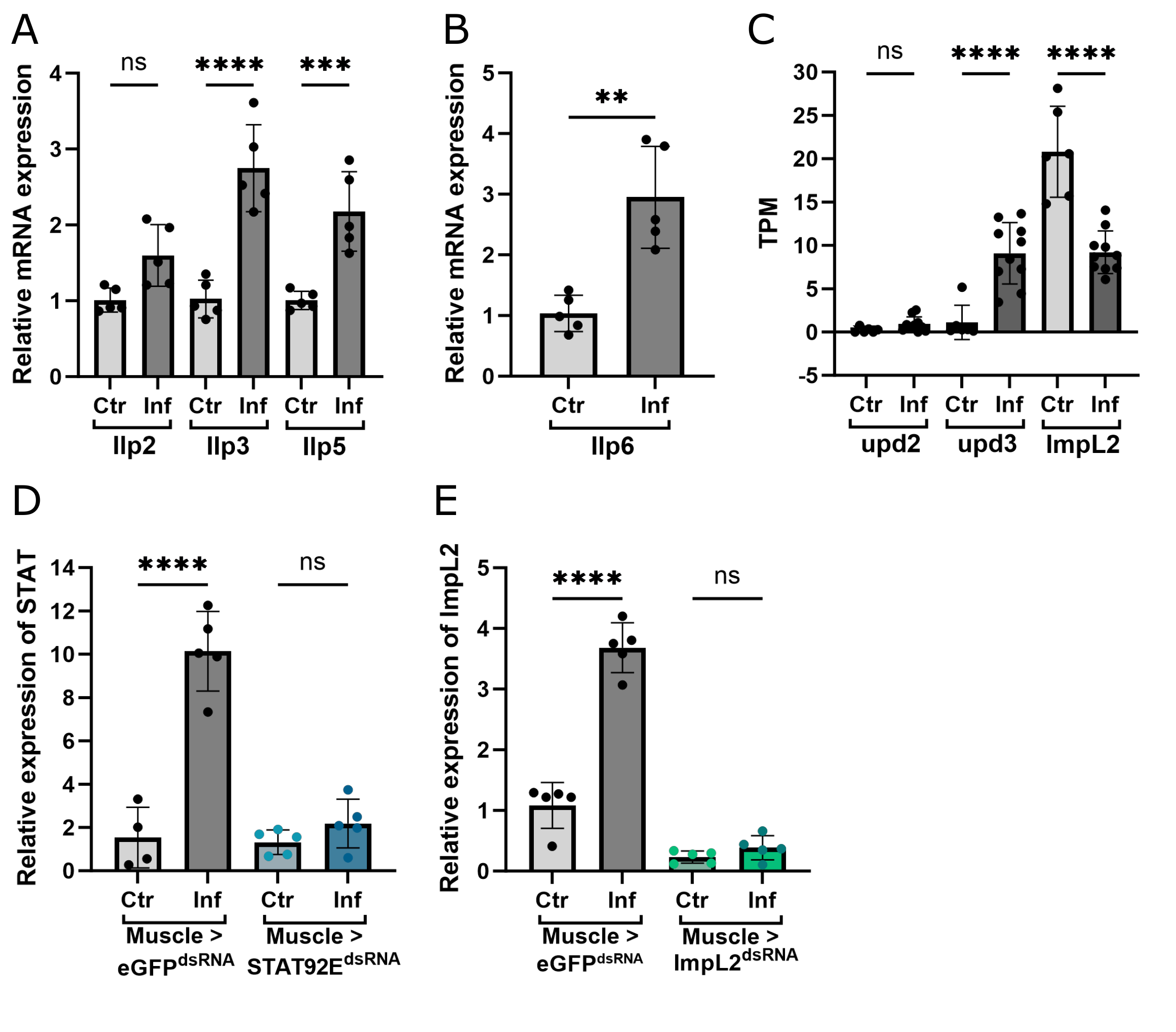

Supplement: Supplementary file 2 — Supplementary Material 2: Supplementary Fig. 2 Expression of Ilps in the brain and fat body. A: Relative expression of Ilp2, Ilp3 and Ilp5 in the central nervous system of control and infected third instar larvae 28 hpi (n = 5). B: Comparative expression of Ilp6 in the fat body of control and infected animals (n = 5). C: Expression of upd2, upd3 and ImpL2 (bulk RNAseq) in hemocytes with and without infection, Y axis shows transcripts per million (TPM) (n = 6–10) D: Expression of STAT with and without infection in Muscle > eGFPdsRNA and Muscle > STAT92EdsRNA animals (n = 4–5). E: Levels of ImpL2 in ImpL2 knockdown animals and controls with and without infection (n = 5). Bars represent mean values, dots represent biological replicates. ns: no significant difference, ** p ≤ 0.01, *** p ≤ 0.001, **** ≤ 0.0001. N represents individual experiments, n represents biological replicates. [file 12964_2024_1575_MOESM2_ESM.tif]
